# Improving Gene-Set Enrichment Analysis of RNA-Seq Data with Small Replicates

**DOI:** 10.1371/journal.pone.0165919

**Published:** 2016-11-09

**Authors:** Sora Yoon, Seon-Young Kim, Dougu Nam

**Affiliations:** 1 School of Life Sciences, Ulsan National Institute of Science and Technology, Ulsan, Republic of Korea; 2 Medical Genomics Research Center, Korea Research Institute of Bioscience & Biotechnology, Daejeon, Republic of Korea; 3 Department of Bioinformatics, University of Science and Technology, Daejeon, Republic of Korea; 4 Department of Mathematical Sciences, Ulsan National Institute of Science and Technology, Ulsan, Republic of Korea; University of Rochester, UNITED STATES

## Abstract

Deregulated pathways identified from transcriptome data of two sample groups have played a key role in many genomic studies. Gene-set enrichment analysis (GSEA) has been commonly used for pathway or functional analysis of microarray data, and it is also being applied to RNA-seq data. However, most RNA-seq data so far have only small replicates. This enforces to apply the gene-permuting GSEA method (or preranked GSEA) which results in a great number of false positives due to the inter-gene correlation in each gene-set. We demonstrate that incorporating the absolute gene statistic in one-tailed GSEA considerably improves the false-positive control and the overall discriminatory ability of the gene-permuting GSEA methods for RNA-seq data. To test the performance, a simulation method to generate *correlated* read counts within a gene-set was newly developed, and a dozen of currently available RNA-seq enrichment analysis methods were compared, where the proposed methods outperformed others that do not account for the inter-gene correlation. Analysis of real RNA-seq data also supported the proposed methods in terms of false positive control, ranks of true positives and biological relevance. An efficient R package (AbsFilterGSEA) coded with C++ (Rcpp) is available from CRAN.

## Introduction

The high-throughput cDNA sequencing technology (RNA-seq) has enabled an efficient and thorough analysis of the transcriptome in the cell [1, 2]. In particular, RNA-seq exhibited much lower background noise than the hybridization-based method (microarray), which resulted in an improved accuracy in quantitating the gene expression [3]. In spite of this advantage, the differential expression (DE) analysis of RNA-seq data between two sample groups has been a non-trivial task because of the varying sequencing depths and RNA compositions in each sample and the discrete nature of the read count data. To address the problem, various normalization methods have been developed to make the gene expression levels comparable between samples [4, 5], and a variety of methods have been developed to test the DE of each gene based on discrete probability models. [6–12].

To interpret the DE analysis result, Gene Ontology (GO) terms or other gene-sets that share common functions have been used to assess the over-representation of a function in the DE genes, which may be called *GO analysis* [13, 14]. Another useful approach is the gene-set enrichment analysis (GSEA) [15]. Unlike GO analysis, GSEA does not use the cutoff threshold to identify the DE genes, but employs the (weighted) Kolmogorov-Smirnov (K-S) statistic to test whether genes contributing to the phenotype are ‘enriched’ in each gene-set. Thereby, GSEA is able to capture the subtle but coordinated changes in a gene-set and has been commonly used to find important pathways or functions in various diseases and cell conditions from microarray data [16–19].

In spite of the power of GSEA, the pathway analysis methods and tools for RNA-seq have only recently been devised based on methods developed for microarray [11, 20–22]. One of the issues in applying GSEA to RNA-seq data is how to normalize the read count data. voom method transforms the read counts into microarray-like data for which most linear-model based methods developed for microarray can be applied [11]. GSAAseqSP tool [21] deploys TMM or DESeq normalization methods [5, 7] which are able to address both the different depths and RNA compositions between samples. Another important issue is the small replicates: Despite the rapid decrease of the sequencing cost, it is still costly for most laboratories and only a few replicates have been produced for each sample condition [23]. Such small replicates prohibit from using the sample-permuting GSEA (GSEA-SP), but force to use the gene-permuting GSEA (GSEA-GP) which results in a great number of false positive gene-sets caused by the inter-gene correlation in the gene expression.

We demonstrate that the *absolute gene statistic* remarkably reduces the false positive rate and improves the overall discriminatory ability (ROC) of the GSEA-GP methods in analyzing RNA-seq read count data. This property has also been shown for microarray data [24]. RNA-seq read counts were modeled and simulated using discrete probability (negative binomial distribution) [6, 25], and a simulation method to generate ‘correlated’ read counts within a gene-set was newly developed to compare the performance of GSEA methods for RNA-seq data. Note that the inter-gene correlation has a critical effect on the performance of gene-set level analysis, but has not been considered so far for the counting data because of the lack of such a simulation method.

For a more accurate analysis, a one-tailed GSEA method that only considers the *positive* deviation in the K-S statistic was devised for the absolute enrichment analysis. Based on this result, we also propose filtering the GSEA-GP results with those obtained from the absolute GSEA-GP to effectively reduce false positives. The performances of the absolute GSEA and its filtering method were demonstrated for simulated and real RNA-seq data.

## Materials and Methods

### Absolute gene-permuting GSEA and filtering

In many RNA-seq experiments, the replicate size is not large enough to carry out GSEA-SP, in which case the GSEA-GP is used instead. However, the gene-permuting method generates a great number of false positives due to the inter-gene correlation in each gene-set [26–30]. Recently, it has been shown that incorporating the absolute gene statistic in GSEA-GP considerably reduces the false positive rate and improves the overall discriminatory ability in analyzing microarray data [24]. Therefore, we tested whether the absolute statistic exhibits a similar benefit in analyzing RNA-seq counting data. In addition to substituting the gene scores with their absolute values (i.e., flipping the sign of negative gene scores) [31], our absolute GSEA is modified as a one-tailed test by considering only the ‘positive’ deviation in the K-S statistic. We have two reasons for this modification. First, simply replacing the gene scores with their absolute values in GSEA can result in a few ‘down-regulated’ gene-sets which are meaningless in an *absolute* enrichment analysis. Removing these down-regulated gene-sets in itself may confer beneficial effect. Our one-tailed absolute GSEA does not yield any down-regulated gene-sets. Second, it provides more accurate background null distribution of gene-set scores: In the conventional GSEA algorithm, the maximum positive and negative deviation values are compared and only the larger absolute value between the two is selected for the gene-set score. This means the minor maximum deviation values are all excluded in constituting the gene-set null distributions. By taking only the positive deviation values (one-tailed K-S statistic), every gene-set participates in constituting the null distribution of absolute gene-set statistic even when the negative deviation is larger. In real RNA-seq data analysis, the one-tailed method tended to be more conservative than the two-tailed method.

#### Gene scores

Four gene scores were considered for normalized read as follows:

1. Moderated *t*-statistic (mod-*t*): A modified two-sampe *t*-statistic
  $$\tilde{t_{i}}=\frac{\mu_{i}^{1}-\mu_{i}^{2}}{\tilde{s_{i}}\sqrt{\upsilon_{i}}}$$
  where $\mu_{i}^{n}$ is the mean read count of *i*th gene, *g*_*i*_ in class *n*, and $\tilde{s_{i}}$ is a shrinkage estimation of the standard deviation of *g*_*i*_. This statistic is useful for small replicate data and is implemented using the limma R package [32, 33]
2. Signal-to-Noise ratio (SNR): The SNR (*S*_*i*_) is calculated as
  $$S_{i}=\frac{\mu_{i}^{1}-\mu_{i}^{2}}{\sigma_{i}^{1}+\sigma_{i}^{2}}$$
  where $\sigma_{i}^{n}$ is the standard deviation of expression values of *g*_*i*_ in class *n*.
3. Zero-centered rank sum (Ranksum): This two-sample Wilcoxon statistic is introduced by Li and Tibshirani [34]. For *g*_*i*_, the rank sum test statistic (*T*_*i*_) is calculated as,
  $$T_{i}=\sum_{j\in C_{1}}R_{ij}-\frac{n_{1}∙(n+1)}{2}$$
  where *R*_*ij*_ is the rank of expression level of *j*^*th*^ sample among all counts of *g*_*i*_, *C*_1_ is a set of sample indexes in the first phenotypic class, *n*_1_ is the sample size of *C*_1_ and *n* is the total sample size. Note that E(*T*_*i*_) = 0.
4. Log fold-change (logFC): Log fold-change (log*FC*_*i*_) for *g*_*i*_ is calculated as
  $${logFC}_{i}=\log_{2}\frac{\mu_{i}^{1}}{\mu_{i}^{2}}$$

#### Absolute GSEA

GSEA algorithm identifies functional gene-sets that show a coordinated gene expression change between given phenotypes from gene expression profiles. Given gene scores, GSEA implements a (weighted) K-S statistic to calculate the enrichment score (ES) of each pre-defined gene-set.

1. Enrichment score: Let *S* be a gene-set and *r*_*i*_ be the gene score of *g*_*i*_. Then, the enrichment score ES(*S*) is defined as the maximum deviation of *p*_*hit*_ – *p*_*miss*_ from zero, that is
  $$ES(S)=\left\{ \begin{array}{l} \underset{i}{\max}\left(p_{hit,i}-p_{miss,i}\right),\;if\;|\underset{i}{\max}\left(p_{hit,i}-p_{miss,i}\right)|\geq |\underset{i}{\min}\left(p_{hit,i}-p_{miss,i}\right)|\\ \underset{i}{\min}\left(p_{hit,i}-p_{miss,i}\right),\;if\;|\underset{i}{\max}\left(p_{hit,i}-p_{miss,i}\right)|<|\underset{i}{\min}\left(p_{hit,i}-p_{miss,i}\right)| \end{array}\right.$$
  where
  $$p_{hit,i}=\sum_{\begin{array}{c} g_{j}\in S\\ j\leq i \end{array}}\frac{{\left|r_{j}\right|}^{q}}{N_{R}},\;p_{miss,i}=\sum_{\begin{array}{c} g_{j}\in S^{c}\\ j\leq i \end{array}}\frac{1}{\left(N-N_{H}\right)},\;N_{R}=\sum_{g_{j}\in S}{\left|r_{j}\right|}^{q}$$
  *N* is the total number of genes in the dataset, *N*_*H*_ is the number of genes included in *S* and *q* is a weighting exponent which is set as one in this study as recommended [15]. (For the classical K-S statistic, *q* = 0)
2. ES for one-tailed absolute GSEA: The absolute GSEA can be simply conducted by replacing the gene scores by their absolute values, but the orders of gene scores are quite different from the original GSEA algorithm in calculating the K-S statistic. For the one-tailed test, only the positive deviation ES(*S*) = max_*i*_(*p*_*hit*,*i*_ – *p*_*miss*,*i*_) (right-tailed K-S statistic) is considered for the gene-set score even when the negative deviation is larger.

Then, the *gene permutations* are applied, and the corresponding ES’s are calculated and normalized for evaluating the false discovery rate of each gene-set [15].

#### Filtering with absolute GSEA

To reduce the false positives in the GSEA-GP, we propose using the absolute GSEA-GP results for filtering false positives from the ordinary GSEA-GP results. In other words, only the gene-sets that are significant in both ordinary and the one-tailed absolute GSEA are considered significant. In this way, more reliable gene-sets with directionality can be obtained. In all the analyses presented in this paper, the same FDR cutoff is applied for both ordinary and absolute methods, but different cutoffs can also be considered for stricter or looser filtering.

### Simulation of the read count data with the inter-gene correlation

Inter-gene correlation in each gene-set critically affects the performance of gene-permuting gene-set analysis methods (a.k.a. competitive analysis) [26, 35]. For microarray data, multivariate normal distributions have been used for modeling the inter-gene correlation [24, 29, 36], which cannot be directly applied for ‘discrete’ read count data. Here, we present a method to simulate read count data involving the inter-gene correlation within each gene-set. *N* = 10,000 genes are considered and the replicate sizes for the test and control groups are *n*_1_ and *n*_2_, respectively.

**Step 1.** Parameter estimation and read count generation: The read count *X*_*ij*_ of ith gene in jth sample has been modeled by an over-dispersed Poisson distribution, called negative binomial (NB) distribution [6, 7, 25] denoted by $X_{ij}∼NB(\mu_{ij},\sigma_{ij}^{2})$ where *μ*_*ij*_ and $\sigma_{ij}^{2}=\mu_{ij}+\varphi_{i}\mu_{ij}^{2}$ are the mean and variance, respectively, and *φ*_*i*_ ≥ 0 is the dispersion coefficient for gene *g*_*i*_. Here, *μ*_*ij*_ = *s*_*j*_*μ*_*i*_, where *s*_*j*_ is the 'size factor' or 'scaling factor' of sample j and *μ*_*i*_ is the expression level of *g*_*i*_. For simplicity, we assume all the size factors *s*_*j*_ = 1 in this simulation study. To obtain realistic parameters, after filtering out genes with less than 10 average counts and normalization, 10,000 genes were randomly selected from the TCGA kidney RNA-seq dataset (denoted as TCGA KIRC) [37], and their mean and tag-wise dispersions were estimated using the edgeR package [25]. The read counts were generated using the R function 'rnbinom' where the inverse of the estimated dispersion *φ*_*i*_ was input as the ‘size’ argument. This method generates read counts that are independent between genes.

**Step 2.** Generation of read count data with the inter-gene correlation: Given a gene-set *S* with *K* genes, the inter-gene correlation can be generated by incorporating a common variable within the gene-set. Let *μ*_*i*_ and *φ*_*i*_, *i* = 1,2,…*K* be the mean and tag-wise dispersion of *g*_*i*_ in the gene-set and *C*_*ij*_ be the read count generated from these parameters (*Step 1*). Let $P_{S}=\left\{p_{1},p_{2},\cdots ,p_{n_{1}{+n}_{2}}\right\}$ be probability values randomly sampled from the uniform distribution U(0,1). Then, for each *g*_*i*_, the probability values in *P*_*S*_ are converted to a read count $C_{ij}^{*}$, *j = 1*,*2*,*…*, *n*_1_+*n*_2_ using the inverse function of the individual gene's distribution *X*_*i*_∼NB(*μ*_*i*_,*φ*_*i*_) such that $p_{j}\approx P(X_{i}\leq C_{ij}^{*})$. In short, $C_{ij}^{*}$ are generated from the common uniform distribution via the gene-wise NB distribution. The 'correlated' read count for *i*th gene in *j*th sample is then obtained by the weighted sum of the original count *C*_*ij*_ and the ‘commonly generated’ count $C_{ij}^{*}$ as follows:

$$M_{ij}:=\left[(1-\alpha )∙C_{ij}+\alpha ∙C_{ij}^{*}\right]$$

where α ∈ [0,1] is the mixing coefficient that determines the strength of the inter-gene correlation and [] rounds the value to the nearest integer. One problem with this count is that its variance is reduced as much as (2α^2^ − 2α + 1) because

$${Var(M}_{ij})\approx {\left(1-\alpha \right)}^{2}∙V(C_{ij})+\alpha^{2}∙V(C_{ij}^{*})=(2\alpha^{2}-2\alpha +1)∙\sigma_{ij}^{2}$$

**Fig 1:**
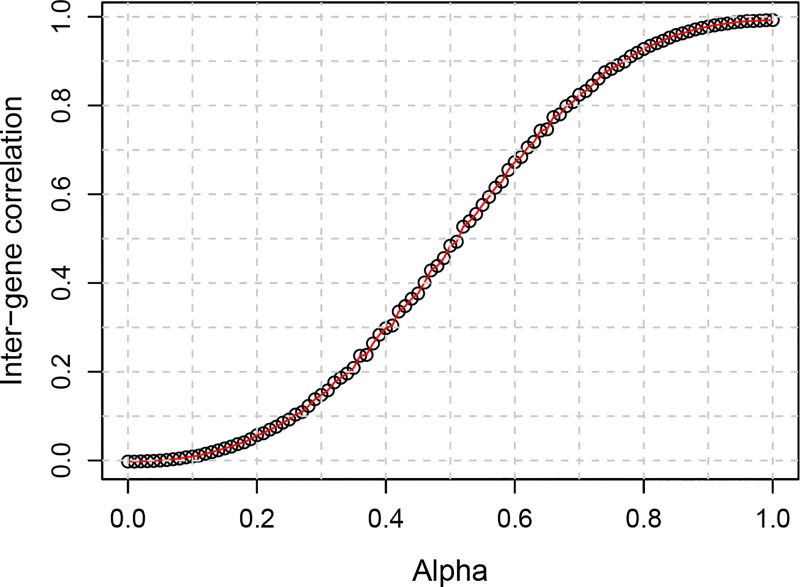
The relationship between the mixing coefficient (alpha) and the average inter-gene correlation.

To remove this factor, we use an inflated dispersion $\varphi_{i}^{\prime }$ derived from the equation

$$(2\alpha^{2}-2\alpha +1)∙(\mu_{i}+\varphi_{i}^{\prime }\mu_{i}^{2})=\mu_{i}+\varphi_{i}\mu_{i}^{2}$$

$$\varphi_{i}\prime =\frac{1+\varphi_{i}\mu_{i}}{\mu_{i}∙(2\alpha^{2}-2\alpha +1)}-\frac{1}{\mu_{i}}$$

instead of *φ*_*i*_ in generating *C*_*ij*_ and $C_{ij}^{*}$. The relationship between *α* and inter-gene correlation is shown in Fig 1.

### Mining PubMed abstracts for scoring biological relevance of gene-sets in each tissue

To assess the biological relevance of gene-sets with the absolute GSEA filtering, a literature-based gene-set score was devised using the PubMed abstracts. Deregulated gene-sets may be featured by their member genes that are closely related to the tissue corresponding to the input data. For a significant gene-set *S*, its relevance with a specific tissue *T* is scored by the log geometric average of the abstract counts as follows:

$$L(S)=\frac{1}{K}\sum_{i=1}^{K}log(A_{T,i})$$ (1)

where *K* is the gene-set size and *A*_*T*,*i*_ is the number of PubMed abstracts where both the keywords related to the tissue *T* and the name of *g*_*i*_ co-occur. The literature mining was conducted using RISmed R package [38].

### Processing RNA-seq data and gene-set size condition

For all the RNA-seq datasets analyzed in this study, raw read counts were normalized by the median method in DESeq [7], and then the lower five percentile of the normalized counts exclusive of the zero counts was used as an offset to stabilize the logFC score of genes with some low expression. Note that the offset has no effect on the other gene scores. The ‘gene-set size’ means the number of genes that are found in both the original gene-set and the RNA-seq dataset. When performing GSEA, the gene-set sizes between 10 and 300 inclusive were used.

### AbsFilterGSEA R package

An efficient R package ‘AbsFilterGSEA’ that implements the GSEA-GP with or without absolute filtering and the absolute GSEA-GP was developed and is available from CRAN [39]. It accepts a raw read count matrix and normalizes it using DESeq median method [7]. It also accepts an already normalized dataset. The core GSEA was coded with C++ and the results were cross-checked with those from the original GSEA R-code [15]. It takes only several seconds to minutes depending on the number of gene-sets tested. The integration of C++ code to the R package was implemented using Rcpp package [40].

## Results

### Comparison of gene-permuting GSEA methods for simulated read count data

To compare the performance of GSEA methods for small replicates, twelve gene-permuting gene-set analysis methods were tested using simulated read count data incorporating the inter-gene correlation as described in Methods. The simulated read count data included 10,000 genes and 100 non-overlapping gene-sets each of which contained 100 genes.

**Fig 2:**
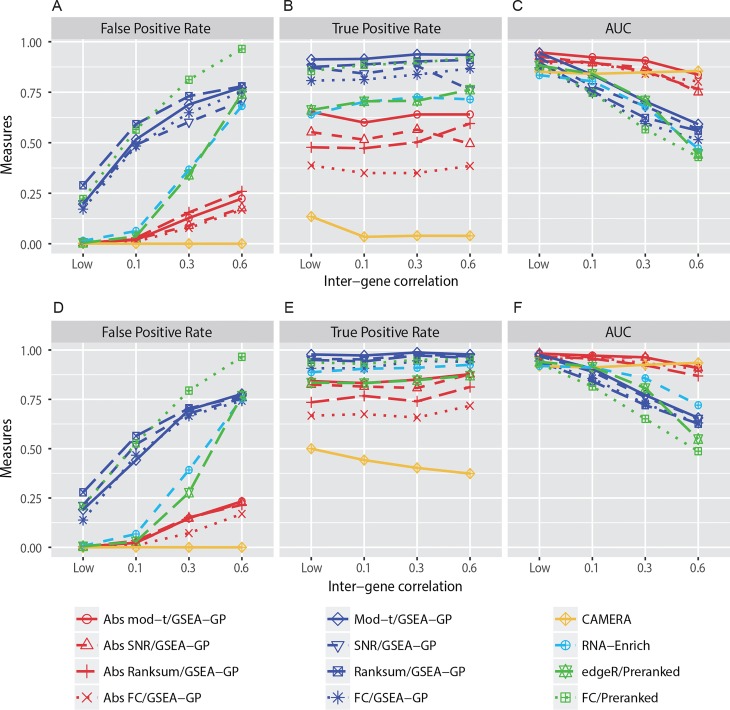
Performance comparison of gene-permuting GSEA methods for simulated read counts. GSEA-GP methods combined with eight gene statistics, (moderated *t*-statistic, SNR, Ranksum, logFC and their absolute versions), Camera combined with voom normalization, RNA-Enrich and two preranked GSEA methods for edgeR *p*-values and FCs were compared for false positive rate, true positive rate and area under the receiver operating curve using simulated read count data with three (A-C) and five replicates (D-F).

First, the false positive rates (FPRs; FDR < 0.1) of the GSEA-GP methods for the four gene statistics (mod-*t*, SNR, Ranksum and FC) and their absolute counterparts were measured using the simulated read count datasets with four different levels of inter-gene correlation, LOW (0~0.05), 0.1, 0.3 and 0.6 within each gene-set. Two, three and five replicates in each sample group were tested and no DE genes were included. This test was repeated twenty times and their average FPRs were depicted in Fig 2A and 2D for three and five replicates, respectively. The result for two-replicate case is available in Figure A in S1 File.

A recently developed competitive method, Camera combined with the voom normalization [29, 41], the bias-adjusted random-set method (RNA-Enrich) [22] as well as two preranked GSEA methods [15] were also compared. The preranked GSEA was implemented using the GSEA R-code [15] where the ranks of genes were determined according to either the *p*-values resulted from the differential expression analysis using edgeR [25] package or the simple absolute fold-changes of the normalized count data. Note that SeqGSEA [42] provides only sample–permuting GSEA which is not useful for small replicates, and GSAAseqSP [43] provides a gene-permuting GSEA method which is virtually the same as GSEA-GP described in this paper (We checked they yielded nearly the same results for the simulated count data). Although it is described that GSAAseqSP uses the absolute gene scores, they are only used for the step-sizes in K-S statistic, and it is far from the ‘absolute’ enrichment analysis.

The FPRs of GSEA-GP for the four ordinary gene statistics and the two preranked methods went up rapidly as the inter-gene correlation was increased. However, the increase rates of FPRs for the four absolute GSEA methods were considerably lower than those for the ordinary statistics. For example, when three replicates were used, even for a moderate inter-gene correlation 0.1, the FPRs for the original statistics were approximately 50% or higher while only a few false positive sets were detected for the absolute methods (1 ~ 3%). Camera yielded no false positives for each correlation level. Overall similar FPR trends were observed with five replicates. RNA-Enrich and the edgeR/preranked methods exhibited relatively better FPRs compared to the GSEA-GP and FC/Preranked methods.

**Fig 3:**
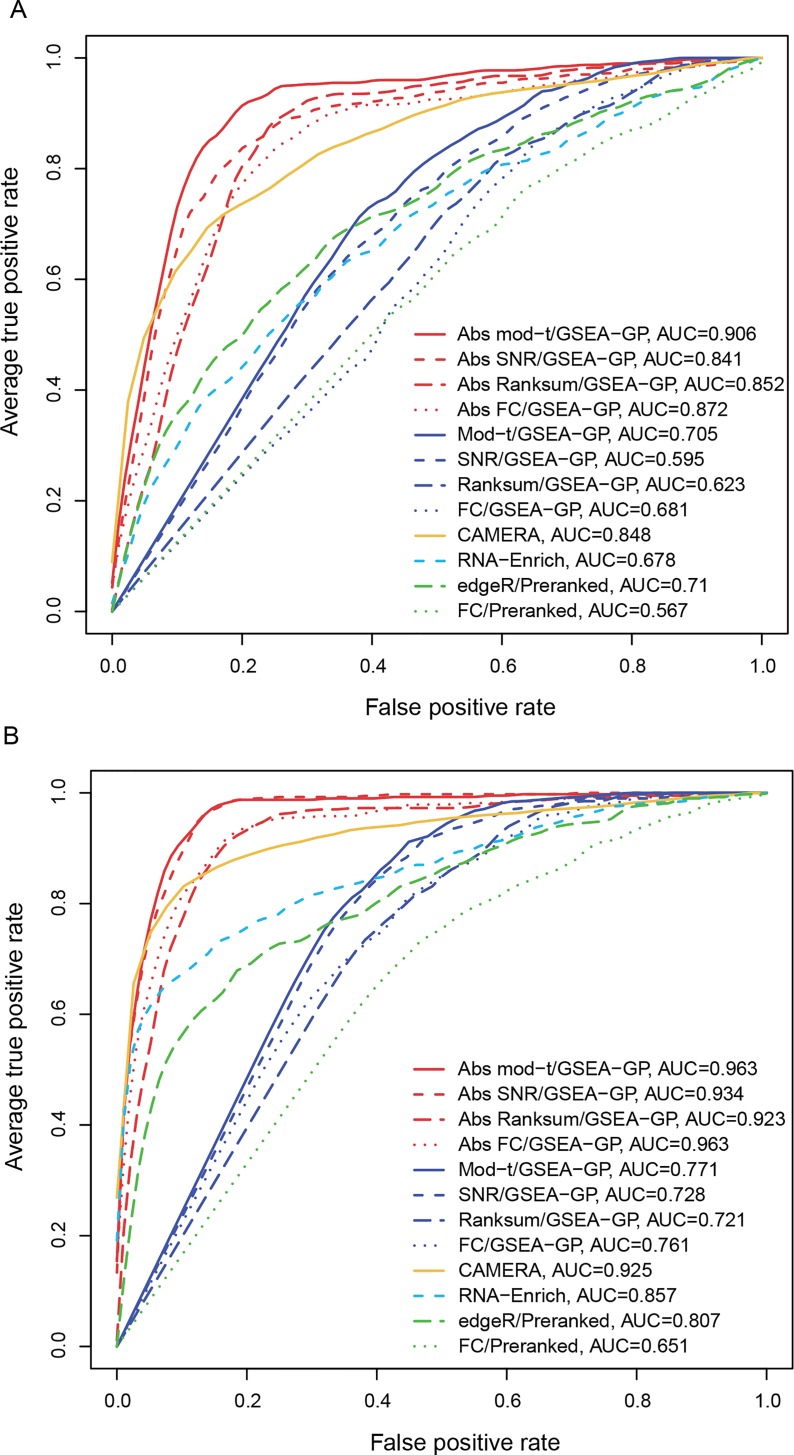
Average receiver operating characteristic (ROC) curves. The average ROC curves (20 repetitions) of the twelve gene-permuting GSEA methods applied to simulation data with the inter-gene correlation of 0.3 for (A) three and (B) five replicate cases.

Next, 20% of the gene-sets (20 gene-sets) in the data generated above were replaced with differentially expressed gene-sets to compare the power (true positive rate) and the overall discriminatory abilities (ROC). These gene-sets included 20~80% (uniformly at random in each gene-set) of DE genes whose mean counts in the test or control group were multiplied by 1.5~2.0 with which the read counts in the corresponding group were regenerated. Only weak inter-gene correlations between 0 and 0.05 were randomly assigned to the DE gene-sets and the four different inter-gene correlation levels were applied for the non-DE gene-sets. The corresponding powers and the area under the ROC curves (AUCs) were then obtained for the twelve methods compared (Fig 2B, 2C, 2E and 2F). The GSEA-GP methods and preranked GSEA with FCs had the highest level of power, but their AUCs rapidly declined as the inter-gene correlation level was increased because of their poor false positive controls. With the inter-gene correlation of 0.6, their performances were close to a random prediction (AUC≈ 0.5). On the other hand, the absolute GSEA-GP methods and Camera were less affected by the inter-gene correlation level and exhibited stable and good AUCs. Among the absolute methods, the mod-*t* gene score resulted in best powers and AUCs. The ROC curves (average of 20 repetitions) of the twelve gene-permuting GSEA methods for the inter-gene correlation 0.3 are illustrated in Fig 3 and Figure B in S1 File.

For the two-replicate data, the FPRs were similar to those of triplicate case, but the powers and AUCs were rather lowered (Figure A in S1 File). While the mod-*t* still exhibited best powers and AUCs among the absolute methods, the power of SNR was considerably lowered, which necessitates the moderated gene statistic in GSEA of small replicate data. Lastly, different inter-gene correlations were randomly assigned for gene-sets in a dataset, and two, three and five replicate cases were tested (Figure A in S1 File). The absolute *mod-t* absolute method still exhibited best AUCs in most cases and exhibited overall similar trends as the identical inter-gene correlation cases.

Overall, these results indicate that the absolute GSEA-GP provides an excellent false positive control and improves the overall discriminatory ability of GSEA-GP. Although the ordinary GSEA-GP methods exhibited best powers, the true positives are overwhelmed by the prohibitively high rate of false positives resulting in very poor ranks of true positives (AUCs). In general, the false positive control and power may be regarded as a tradeoff between different methods, but the overall gain is represented by ROC analysis which demonstrated a clear improvement with the absolute GSEA methods. Compared with Camera, the absolute methods yielded a little more false positives, but exhibited better power and overall discriminatory ability (correlation≤0.3). For small replicate datasets, not all of the true positives may be prioritized perfectly by any method, but it would be important to discern some of the truly altered gene-sets reliably. The proposed absolute GSEA approach provides a simple and reasonable solution for this purpose. However, if minimizing true negatives is of main interest, we recommend investigating all the ‘significant’ gene-sets (typically hundreds) resulted from the ordinary GSEA-GP or generating more samples for GSEA-SP.

### Comparison of GSEA methods for RNA-seq data

The performances of GSEA methods were compared for published RNA-seq datasets in several aspects. First, two RNA-seq datasets denoted by Pickrell and Li data, respectively, were analyzed for comparing power and accuracy as follows:

The Pickrell data were generated from the lymphoblastoid cell lines of 69 unrelated Nigerian individuals (29 male and 40 female) [44]. To analyze the chromosomal differences in expression between male and female, MSigDB C1 (cytogenetic band gene-sets) [45–47] was used for analysis. The GSEA-SP with SNR gene score was applied for the total dataset which resulted in two significant gene-sets ‘chryq11’ (FDR = 0.00143) and ‘chrxp22’ (FDR = 0.0514) both of which were sex-specific. These two gene-sets were significantly up-regulated in male and female groups, respectively. Since the GSEA-SP controls the false positives well, these two gene-sets were regarded as true positives. Then, five samples were randomly selected from each group to constitute a small replicate dataset and GSEA-GP methods with or without absolute filtering, Camera, edgeR/Preranked methods were compared for this small replicate dataset. This process was repeated ten times. Using mod-*t* and logFC as the gene scores, on average, the GSEA-GP yielded 48.2 and 20.4 significant (FDR<0.25) gene-sets including 1.6 and 1.1 true positives, respectively. On the other hand, GSEA-GP with the absolute filtering resulted in only 2.6 and 3.5 significant gene-sets which included 1.11 and 1 true positives for the mod-*t* and logFC gene scores, respectively. For these five-replicate datasets, Camera did not detect any significant gene-set, and the edgeR/Preranked detected as many as 137.4 which included 1.8 true positives. This result implies that the absolute filtering method effectively reduces the false positives resulted from GSEA-GP while maintaining a good statistical power.

A similar trend was observed with the Li dataset. The Li data [48] were generated from LNCaP cell lines with three samples treated with dihydrotestosterone (DHT) and four control samples. The MSigDB C2 (curated gene-set) was used for analysis and the six gene-sets containing the term ‘androgen’ were regarded as potential true positives since DHT is a kind of androgen, though there can be other truly altered gene-sets. When the GSEA-SP with mod-*t* and logFC gene score was applied for this small replicate dataset, as expected, only one and no 'androgen' gene-set was significant (FDR<0.1), respectively. On the other hand, GSEA-GP with mod-*t* and logFC gene scores yielded as many as 187 and 569 significant gene-sets, respectively, which included four 'androgen' gene-sets with FDR≤0.0067. When the absolute filtering was applied, the numbers of significant gene-sets were dramatically reduced to *eight* (Table 1) and 242, which included three and four 'androgen' gene-sets, respectively. Of note, the top three gene-sets were ‘androgen’ terms for the mod-*t* score. The absolute GSEA filtering with SNR score provided a similar result. Camera detected only two 'androgen' gene-sets within 101 significant gene-sets with FDR = 0.00836 and 0.0195, respectively. RNA-Enrich and edgeR/Preranked were so sensitive for this dataset that 1108 and 782 sets were significant (FDR<0.1). RNA-Enrich and edgeR/Preranked detected *four* and three androgen terms within top 52 and 91 gene-sets.

**Table 1:** Significant gene-sets detected by the absolute GSEA-GP filtering (FDR<0.1) with the mod-*t* score (DHT-treated and control LNCaP cell line).

**Gene-set name**	**FDR**	**Literature Score**
**NELSON_RESPONSE_TO_ANDROGEN_DN**	**0**	**2.15**
**NELSON_RESPONSE_TO_ANDROGEN_UP**	**0**	**1.87**
**WANG_RESPONSE_TO_ANDROGEN_UP**	**1.63 × 10**^**−4**^	**1.37**
**PIONTEK_PKD1_TARGETS_UP**	2.79 × 10^−4^	1.78
**REACTOME_AMINO_ACID_SYNTHESIS_AND_INTERCONVERSION_TRANSAMINATION**	2.27 × 10^−2^	1.94
**WANG_RESPONSE_TO_FORSKOLIN_UP**	3.02 × 10^−2^	1.42
**VALK_AML_CLUSTER_11**	5.12 × 10^−2^	1.17
**HUPER_BREAST_BASAL_VS_LUMINAL_UP**	4.40 × 10^−2^	1.53

Overall, the results for real data analysis were concordant with the simulation results. GSEA-GP yielded a large number of significant gene-sets most of which seemed to be false positives. The absolute filtering method considerably reduced false positives at the cost of small loss of power. Camera exhibited a strict false positive control, but its power was relatively weak. In particular, the absolute filtering with mod-*t* score exhibited a high precision and a good power in both datasets. The absolute filtering with the two-tailed absolute GSEA-GP yielded a little more liberal results which are available from Supporting Information.

### Effects of the absolute filtering on false positive control and biological relevance

Here, the effects of the absolute filtering were analyzed for real data in two other aspects. The first one is the false positive rate as investigated with the variance inflation factor (VIF). The FPR of a competitive gene-set analysis method is known to be determined by VIF which is defined as:

$$Var(gene\;set\;statistic)={Var}_{i.i.d.}(gene\;set\;statistic)\times VIF$$

where Var_i.i.d._ is the variance of a gene-set statistic under the assumption that genes in each gene-set have independent expression values. For a linear gene-set statistic, the VIF is explicitly represented as a function of the gene-set size (*K*) and the average inter-gene correlation ($\bar{\rho }$) [29, 49] as follows:

$$VIF=1+(K-1)\bar{\rho }$$ (2)

To compare the FPRs of the GSEA-GP and the absolute GSEA-GP methods, VIF distributions (2) of the significant gene-sets were compared for two TCGA RNA-seq datasets (KIRC and BRCA tumor vs. normal) [50]. These datasets comprise a large number of cancer and normal samples (144 for KIRC and 216 for BRCA in total, respectively) with which the average inter-gene correlation can be reliably estimated. In each dataset, five cancer samples and five normal samples were randomly drawn to constitute a small replicate dataset, to which GSEA-GP was applied for the MSigDB C2 curated gene-sets using the gene scores logFC and absolute logFC, respectively. Then, the VIFs were compared between two classes of significant gene-sets as follows: One is the gene-sets that are significant only in the ordinary GSEA-GP (class A) and the other is those that are significant in both the ordinary and absolute GSEA-GP methods (class B). Note that the total samples in each dataset were used to calculate $\bar{\rho }$. This process was repeated ten times and the corresponding VIF distributions were compared (Figure C in S1 File). In all cases, VIFs of class B were significantly smaller than those for class A which implies smaller FPRs in the absolute GSEA-GP method. All the ten randomly drawn sub-datasets exhibited significantly smaller VIFs in class B in both the TCGA datasets. (Wilcoxon ranksum p-value<0.05; smallest p-value 9.77E-27 for KIRC and 7.56E-33 for BRCA dataset). This indicates the absolute filtering method substantially reduces the false positives in real data analysis.

The second aspect is the tissue-specific relevance score (1). As the above case, five samples were randomly selected from each group of the KIRC and BRCA datasets ten times, and the literature relevance scores between the class A and B sets were compared (Figure C in S1 File). As a result, for all the ten sub-datasets, the relevance scores in class B were significantly larger for both the KIRC and BRCA datasets (smallest p-value: 1.99E-17 and 2.02E-19, respectively).

In addition, the ratios of cancer-related gene-sets (defined as those sets containing one of following keywords such as ‘cancer’, ‘tumor’ and ‘carcinoma’ in their names) were significantly higher in class B compared to those of class A. On average, the ratio of cancer-related terms in class A and class B were 10.0% and 16.3% in KIRC, and 12.7% and 20.3% in BRCA datasets, respectively (Figure C in S1 File). These results indicate that the absolute filtering method tends to result in more reliable and biologically relevant gene-sets.

## Discussion

Since the advent of RNA-seq technology until recently, various methods to identify DE genes from the RNA-seq read count data have been developed [6, 25, 41, 51]. One notable feature shared by DE analysis methods is that they yield quite a number of DE genes. RNA-seq is known to provide a much improved resolution in quantitating gene expression compared to that of microarray [2], which may have increased the sensitivity of DE analysis for RNA-seq data.

With the increased resolution and sensitivity, the pathway analysis or GSEA are expected to play a crucial role in genomic studies with their ability to detect the ‘subtle but coordinated’ changes in a gene-set [15]. However, in many cases, only GO analysis has been applied for interpreting RNA-seq data [52]. The low application rate of pathway analysis or GSEA for RNA-seq may be ascribed to the lack of tools that are specifically designed for RNA-seq data. The popularly used GSEA software [15] developed for microarray analysis can be used for RNA-seq data by normalizing the read count data ‘appropriately’ or simply applying the gene-permuting method (preranked GSEA) after ranking the gene differential scores using another software (e.g. edgeR or DESeq).

Since the majority of RNA-seq experiments have generated only small replicates, the preranked GSEA methods were often used for function and pathway analysis. However, gene-permuting methods usually result in a great number of false positives due to the inter-gene correlation whatever the replicate sizes are. To date, Camera [29] has been the only method to control the false positive gene-sets caused by the inter-gene correlation in analyzing small replicate read count data, but its statistical power was quite weak. In this study, we showed one-tailed absolute GSEA manifests an excellent false positive control and a good statistical power for analyzing small replicate RNA-seq data. For absolute GSEA, it is natural to consider the one-tailed test, while the conventional GSEA applies two tailed test. Simply flipping the negative gene score is still a two tailed test, so we devised one-tailed version for the absolute enrichment by taking only the positive deviation in K-S statistic. When we compared one-tailed and two-tailed absolute GSEA in simulation tests, both of the methods exhibited nearly the same performances and were effective in reducing false positives. This is not an unexpected result because the absolute GSEA already have larger positive deviations than their negative counterparts in most gene-sets. However, when we compared the two methods in the two real RNA-seq datasets (Pickrell and Li data), the one-tailed method seemed to be more conservative.

To compare the performance of GSEA methods, read count data incorporating the inter-gene correlation were newly designed and simulated. It is crucial to consider the inter-gene correlation in evaluating gene-set analysis methods. The analysis results for the simulated and RNA-seq data commonly demonstrated the effectiveness of the suggested method. As such, the method and tool presented in this paper may facilitate the pathway analysis of RNA-seq data with small replicates.

## Supporting Information

S1 File Contains Supporting Figures A, B and C and Comparison of one-tailed and two-tailed absolute GSEA results.
(PDF)
