# Supplementary material for: Improving Gene-Set Enrichment Analysis of RNA-Seq Data with Small Replicates
Source: PLoS One. 2016 Nov 9;11(11):e0165919. doi: 10.1371/journal.pone.0165919 (PMC5102490; doi:10.1371/journal.pone.0165919)
Supplement: S1 File — Contains Supporting Figures A, B and C and Comparison of one-tailed and two-tailed absolute GSEA results. (PDF) [file pone.0165919.s001.pdf]

Supporting Information

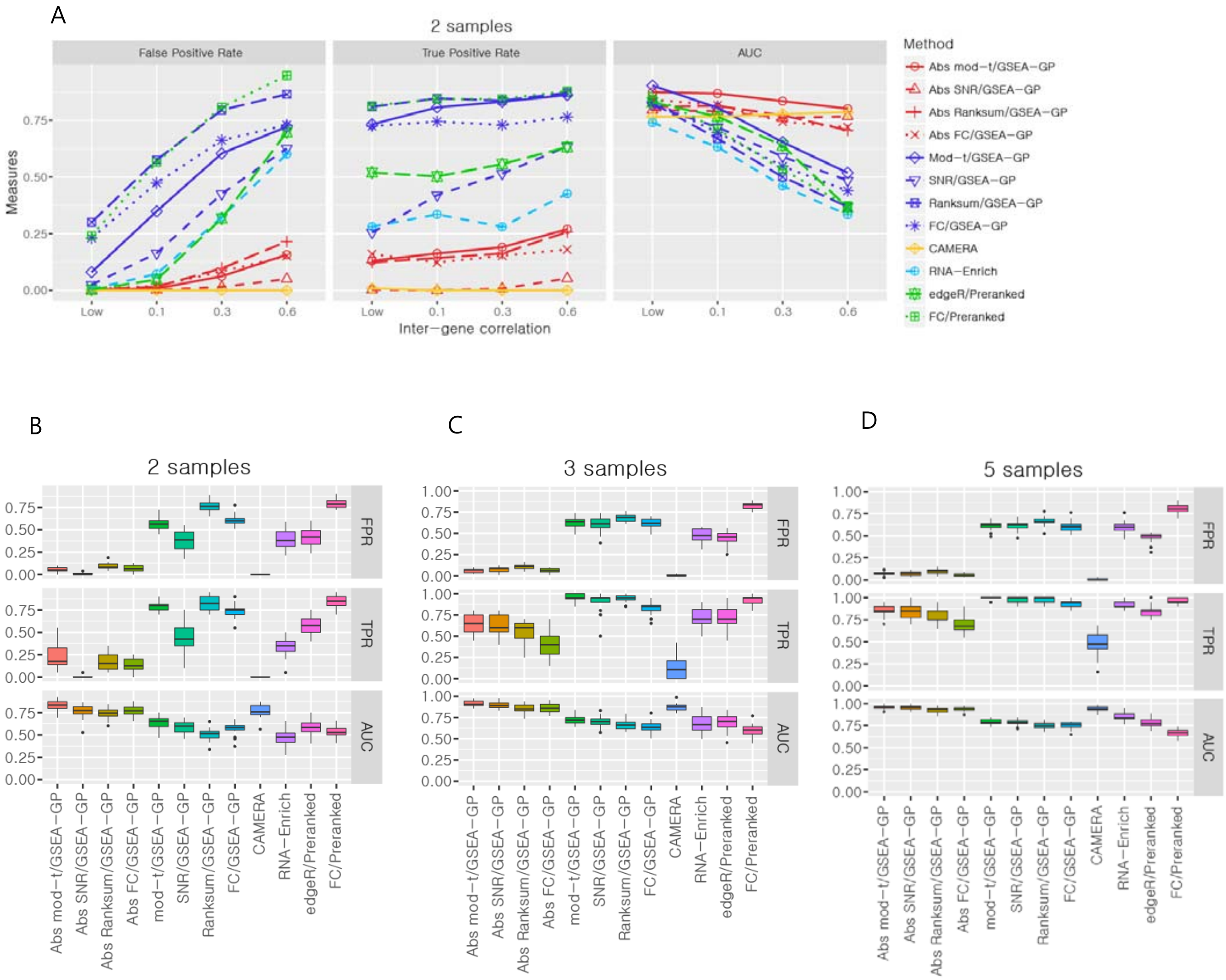

Figure A. Performance comparison of gene-permuting GSEA methods for simulated read counts. GSEA-GP methods combined with eight gene statistics, (moderated t-statistic, SNR, Ranksum, logFC and their absolute values), Camera combined with voom quantile normalization, RNA-Enrich and two preranked GSEA methods for edgeR p-values and FCs were compared for false positive rate, true positive rate and area under receiver operating curve (A) by increasing the inter-gene correlation of simulated read count data composed of two replicates. (B) Simulation results for data with mixture of gene-sets with various inter-gene correlations (0~0.6) for two, (C) three, (D) and five replicates

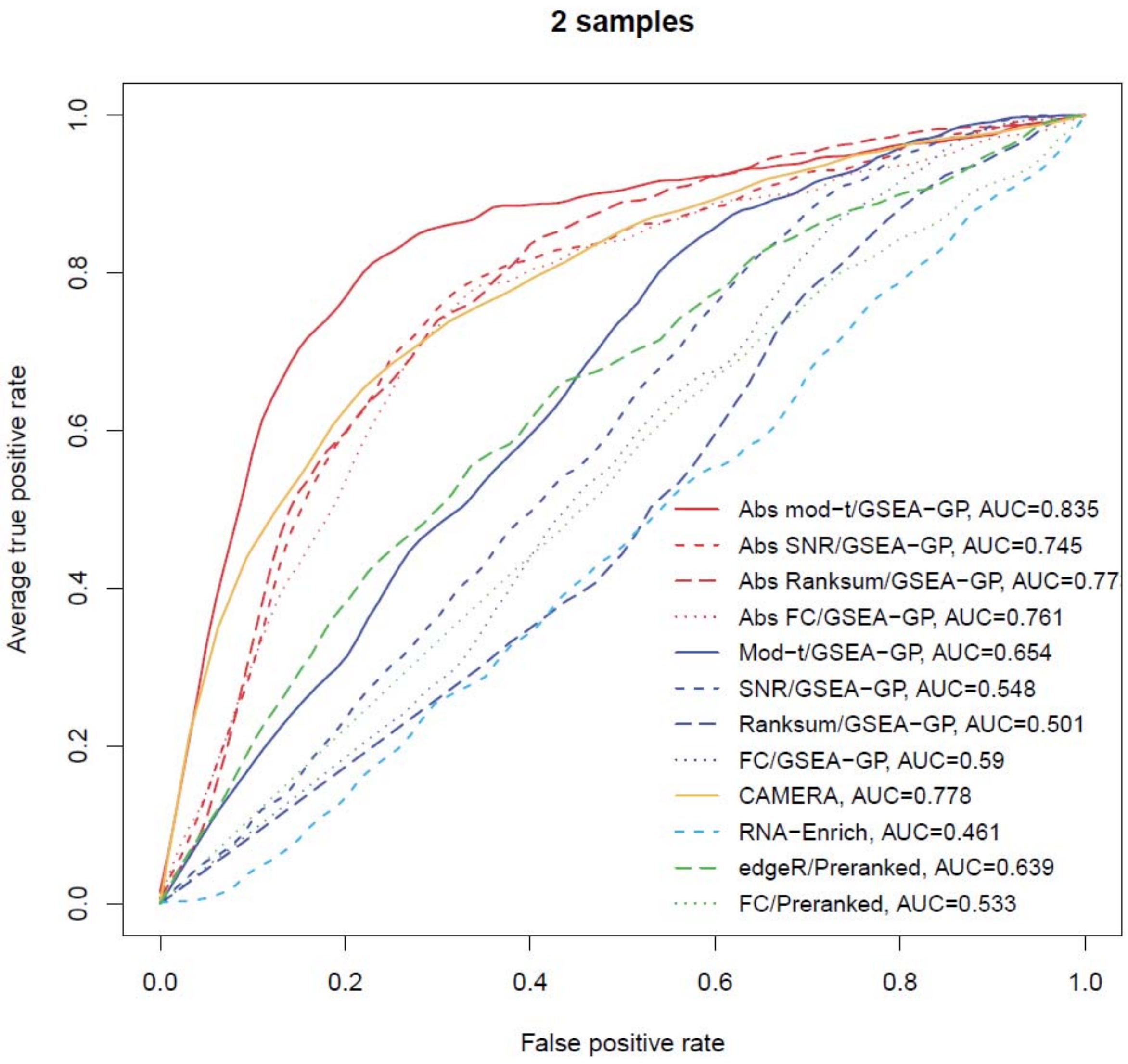

Figure B. Average receiver operating characteristic (ROC) curves for two sample cases. The average ROC curves of the twelve gene-permuting GSEA methods applied to simulation data with inter-gene correlation 0.3 and two replicates.

A

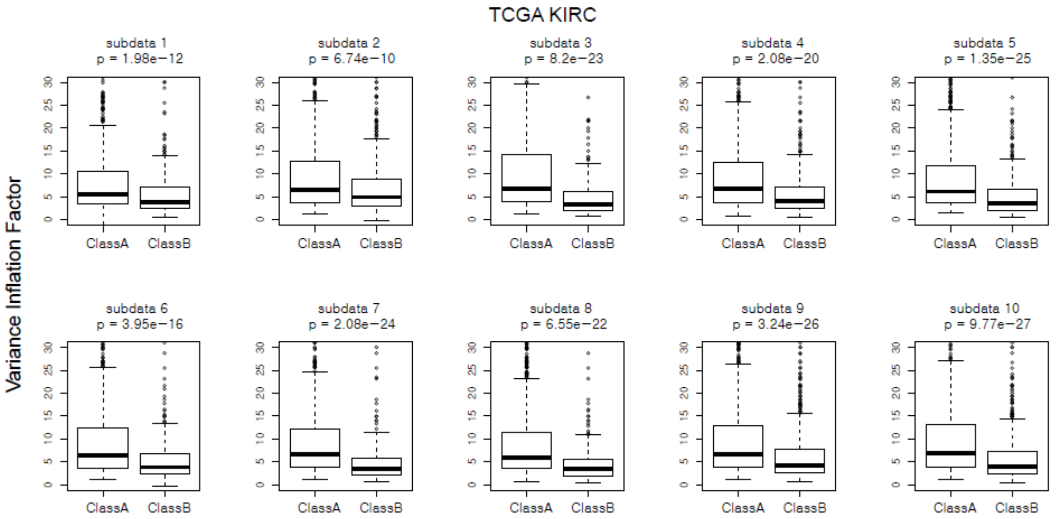
TCGA KIRC
Variance Inflation Factor
subdata 1
p = 1.98e−12
subdata 2
p = 6.74e−10
subdata 3
p = 8.2e−23
subdata 4
p = 2.08e−20
subdata 5
p = 1.35e−25
subdata 6
p = 3.95e−16
subdata 7
p = 2.08e−24
subdata 8
p = 6.55e−22
subdata 9
p = 3.24e−26
subdata 10
p = 9.77e−27
ClassA
ClassB

B

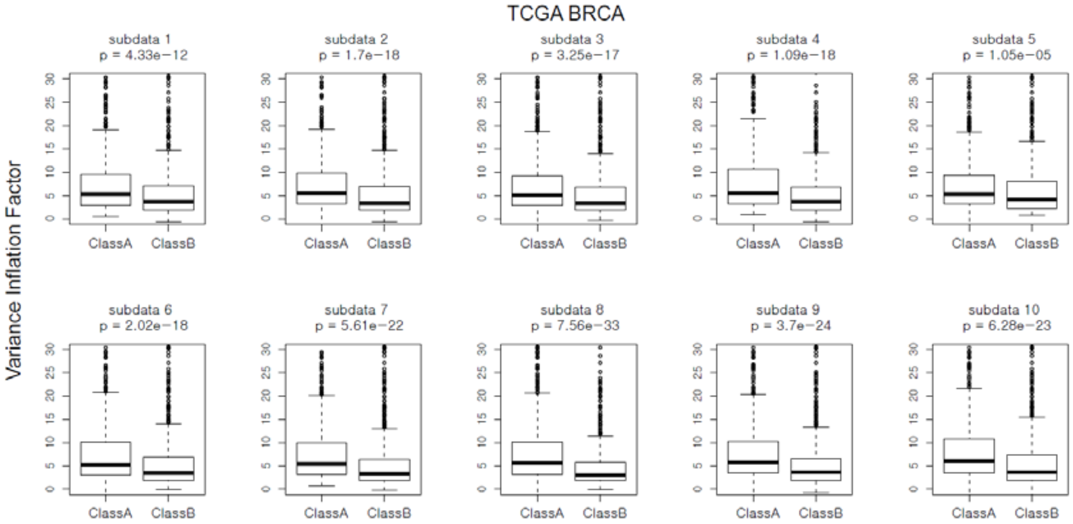
TCGA BRCA
Variance Inflation Factor
subdata 1
p = 4.33e−12
subdata 2
p = 1.7e−18
subdata 3
p = 3.25e−17
subdata 4
p = 1.09e−18
subdata 5
p = 1.05e−05
subdata 6
p = 2.02e−18
subdata 7
p = 5.61e−22
subdata 8
p = 7.56e−33
subdata 9
p = 3.7e−24
subdata 10
p = 6.28e−23
ClassA
ClassB

C

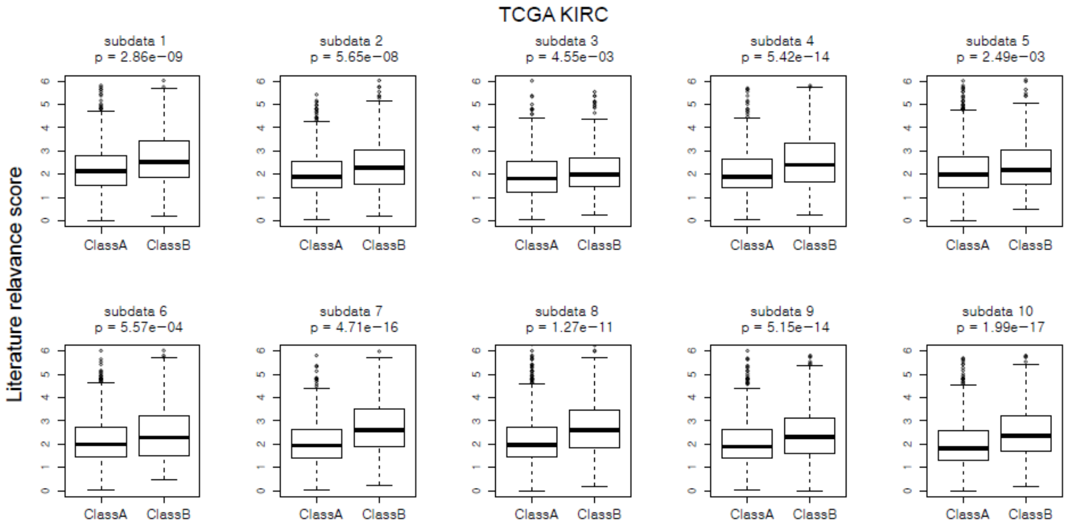
TCGA KIRC
Literature relavance score
subdata 1
p = 2.86e−09
subdata 2
p = 5.65e−08
subdata 3
p = 4.55e−03
subdata 4
p = 5.42e−14
subdata 5
p = 2.49e−03
subdata 6
p = 5.57e−04
subdata 7
p = 4.71e−16
subdata 8
p = 1.27e−11
subdata 9
p = 5.15e−14
subdata 10
p = 1.99e−17
ClassA
ClassB

D

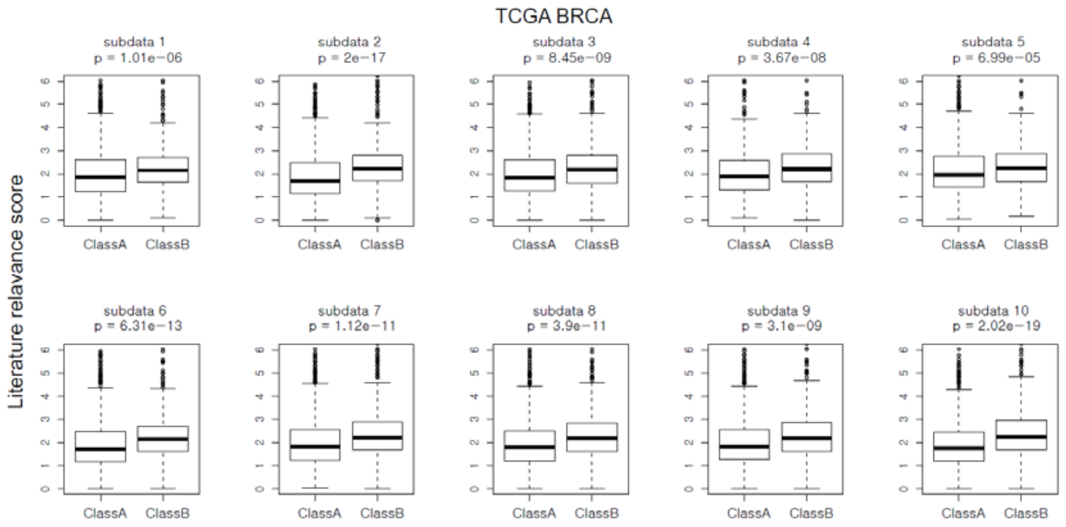

E

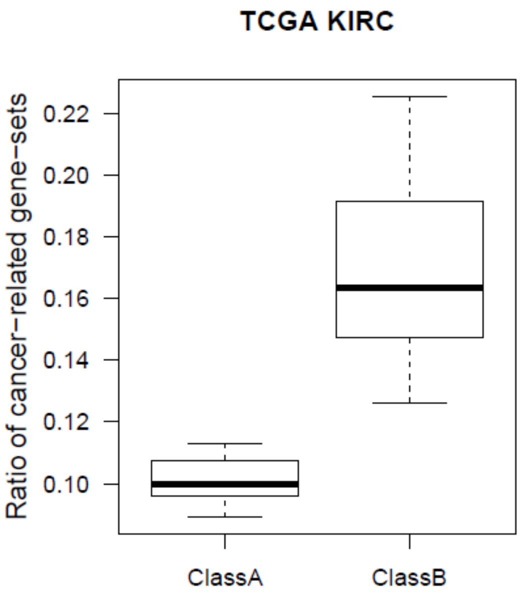

F

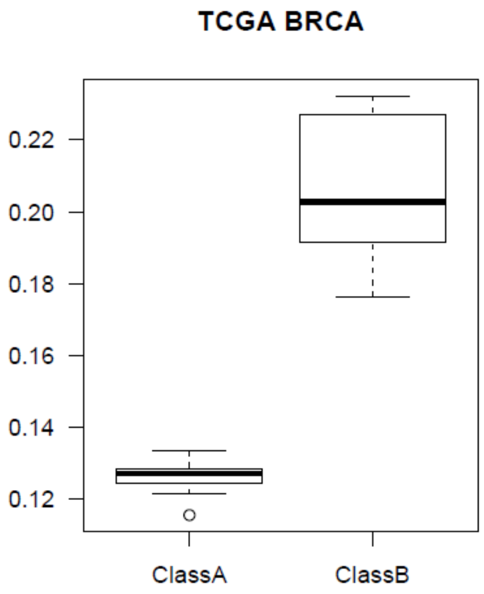

Figure C. The effects of absolute gene-permuting GSEA Five tumor and normal samples were randomly selected from TCGA KIRC and BRCA RNA-seq datasets, and the original and absolute GSEA-GP were performed for each sub-sampled dataset. (A, B) The distributions of variance inflation factor and (C, D) literature score of 'significant' gene-sets (FDR<0.25) were plotted for the ClassA and ClassB (see the main text), and their difference was assessed using Wilcoxon's ranksum test. This process was repeated ten times. (E, F) In addition, the ratio of gene-sets containing terms such as 'cancer', 'tumor', or 'carcinoma' were compared between class A and B.

Comparison of one-tailed and two-tailed absolute GSEA results

We compared the filtering results by one-tailed and two-tailed absolute GSEA in analyzing Pickrell [1] and Li [2] data. Two-tailed absolute GSEA generated more significant gene-sets than one-tailed absolute GSEA. For example, the GSEA-GP with one-tailed absolute filtering for Pickrell data (gene score: moderated-t) resulted in 2.6 significant gene sets (FDR<0.25) including one true term (chryq11) on average, while that of two-tailed filtering yielded 3.3 significant gene sets including one true term on average. When logFC was used as gene score, the one-tailed and two-tailed absolute filtering produced 3.5 and 3.7 significant terms, respectively, including one true term.

Similar result was observed for the Li data. The GSEA-GP with one-tailed absolute filtering detected 8 significant gene sets (FDR<0.1) including three 'androgen'-related gene sets as shown in the Table 1. However, when the two-tailed absolute filtering was applied, it detected 14 significant gene sets including the same three androgen-related terms. When logFC was used as the gene score, the one-tailed and two-tailed absolute filtering detected 242 and 256 significant terms, respectively, and both cases included four androgen-related terms. These results imply that one-tailed absolute GSEA yields a little more conservative results.

1. Pickrell JK, Marioni JC, Pai AA, Degner JF, Engelhardt BE, Nkadori E, et al. Understanding mechanisms underlying human gene expression variation with RNA sequencing. Nature. 2010;464(7289):768-72. doi: 10.1038/nature08872. PubMed PMID: WOS:000276205000047.
2. Li H, Lovci MT, Kwon YS, Rosenfeld MG, Fu XD, Yeo GW. Determination of tag density required for digital transcriptome analysis: Application to an androgen-sensitive prostate cancer model. P Natl Acad Sci USA. 2008;105(51):20179-84. doi: 10.1073/pnas.0807121105. PubMed PMID: WOS:000261995600035.
